# Phenolics from *Garcinia mangostana* alleviate exaggerated vasoconstriction in metabolic syndrome through direct vasodilatation and nitric oxide generation

**DOI:** 10.1186/s12906-016-1340-5

**Published:** 2016-09-13

**Authors:** Hossam M. Abdallah, Hany M. El-Bassossy, Gamal A. Mohamed, Ali M. El-halawany, Khalid Z. Alshali, Zainy M. Banjar

**Affiliations:** 1 Department of Natural Products, Faculty of Pharmacy, King Abdulaziz University, Jeddah, 21589 Saudi Arabia; 2 Department of Pharmacognosy, Faculty of Pharmacy, Cairo University, Cairo, 11562 Egypt; 3 Department of Pharmacology, Faculty of Pharmacy, King Abdulaziz University, Jeddah, 21589 Saudi Arabia; 4 Department of Pharmacology, Faculty of Pharmacy, Zagazig University, Zagazig, Egypt; 5 Department of Pharmacognosy, Faculty of Pharmacy, Al-Azhar University, Assiut Branch, Assiut, 71524 Egypt; 6 Department of Medicine, Faculty of Medicine, King Abdulaziz University, Jeddah, 21589 Saudi Arabia; 7 Department of Clinical Biochemistry, Faculty of Medicine, King Abdulaziz University, Jeddah, 21589 Saudi Arabia

**Keywords:** Metabolic syndrome, *Garcinia mangostana*, Relaxation, Benzophenone, Flavonoids

## Abstract

**Background:**

Exaggerated vasoconstriction plays a very important role in the hypertension, a major component of metabolic syndrome (MetS). In the current work, the potential protective effect of methanol extract of fruit hulls of *Garcinia mangostana *L. on the exaggerated vasoconstriction in MetS has been investigated. In addition, the bioactive fraction and compounds as well as the possible mechanism of action have been illustrated.

**Methods:**

The effect of methanol extract of *G. mangostana* (GMT) fruit hulls on the vascular reactivity of aorta isolated from animals with MetS was investigated through bioassay-guided fractionation procedures. GMT was partitioned with chloroform (I) and the remaining mother liquor was fractionated on a Diaion HP-20 with H_2_O, 50 and 100 % methanol to give fractions II, III, and IV, respectively. The effect of total extract (GMT), bioactive fraction and the bioactive compounds on the vasoconstriction were examined in aortae isolated from animals with MetS by incubation for 30 min before exposing aortae to cumulative concentrations of phenylephrine (PE). The direct relaxant effect was also examined by adding cumulative concentrations of the bioactive fraction and its bioactive compounds to PE precontracted vessels. In addition, aortic nitric oxide (NO) and reactive oxygen species (ROS) production was investigated.

**Results:**

Bioassay-guided fractionation of GMT revealed isolation of garcimangosone D (**1**), aromadendrin-8-C-*β*-D-glucopyranoside (**2**), 2,4,3′-trihydroxy benzophenone-6-O-*β*-D-glucopyranoside (**3**), maclurin-6-O-*β*-D-glucopyranoside (rhodanthenone) (**4**), epicatechin (**5**), and 2,3′,4,5′,6-pentahydroxy benzophenone (**6**). Only compounds **2**, **4**, and **5** significantly alleviated the exaggerated vasoconstriction of MetS aortae and in the same time showed significant vasodilation of PE pre-contracted aortae. To further illustrate the mechanism of action, the observed vasodilation was completely blocked by the nitric oxide (NO) synthase inhibitor, Nω-nitro-L-arginine methyl ester hydrochloride and inhibited by guanylate cyclase inhibitor, methylene blue. However, vasodilation was not affected by the potassium channel blocker, tetraethylammonium or the cyclooxygenase inhibitor, indomethacin. In addition, compounds **2**, **4**, and **5** stimulated NO generation from isolated aortae to levels comparable with acetylcholine. Furthermore, **4** and **5** inhibited reactive oxygen species generation in MetS aortae.

**Conclusion:**

The phenolic compounds **2**, **4**, and **5** ameliorated the exaggerated vasoconstriction in MetS aortae through vasodilatation-NO generation mechanism.

**Electronic supplementary material:**

The online version of this article (doi:10.1186/s12906-016-1340-5) contains supplementary material, which is available to authorized users.

## Background

Metabolic syndrome (MetS) is considered as a major worldwide problem that is characterized by hypertension, hyperinsulinemia and obesity. This syndrome is affecting more than quarter of the world population, due to lack of physical activity and high calorie nutrition [1]. People affected by metabolic syndrome are in high risk of developing cardiovascular complications [2]. This is attributed to the effect of hyperglycaemia and oxidative stress on vascular biology [3]. Vascular endothelia play a major role in maintaining cardiovascular homeostasis through releasing a number of mediators that regulate platelet aggregation, coagulation, fibrinolysis, and vascular tone [4]. Hyperglycemia causes vascular damage in different cells of vascular cell wall leading to endothelial dysfunction and reduction in NO production that give rise to vasoconstriction [5]. Therefore, MetS is associated with changes in vascular responsiveness to vasoconstrictors and vasodilators. The changes in vascular reactivity are responsible for the development of many vascular complications [6]. Consequently, searching for drugs that have the ability to overcome endothelial dysfunction will help in the treatment of diabetic complications.

Herbal drugs are commonly used worldwide due to its high efficacy, few side effects, and relatively low cost. Many plants and its active constituents have been reported for their antidiabetic activity [7]. Some phenolic compounds were reported to have relaxant effect on vasoconstriction [8, 9]. Mangosteen is used traditionally throughout Southeast Asia for preventing some diseases including hypertension,, obesity, and diabetic complications [10]. Moreover, it revealed an antidiabetic effect through α-glucosidase inhibition [11]. Also, the fruit causes a decrease in body mass index (BMI) indicating its possible anti-obesity effect. However, the fruit juice has shown anti-obesity potential, accordingly, more detailed studies are required to confirm its efficacy in the prevention and/or treatment of obesity and diabetes [10]. In addition, mangosteen showed a remarkable vaso-relaxant effect on isolated rat aorta [12] and inhibited advanced glycation end products (AGEs) formation at the levels of Amadori product and protein aggregation through saving protein thiol [13]. The phytochemical screening of *G. mangostana* revealed the presence of phenolic compounds, including prenylated xanthones, benzophenones, flavonoids, and anthocyanins [13–15].

Current study aims at the examination of the potential protective effect of methanol extract of *G. mangostana* (GMT) fruit hulls on the exaggerated vasoconstriction in MetS aortae. In addition; the main bioactive fraction and compounds, as well as the possible mechanism of action will be determined.

## Methods

### General

Electron spray ionization mass (ESIMS) was recorded on an LCQ DECA mass spectrometer (Thermo Finnigan, Bremen, Germany) coupled with an Agilent 1100 HPLC using photodiode array detector. NMR spectra were recorded on a Bruker DRX-400 MHz Ultrashield spectrometer (Bruker BioSpin, Billerica, MA, USA). CD_3_OD was used as a solvent, and TMS as the internal reference. Pre-coated thin layer chromatography (TLC) plates; silica gel 60 F_254_ were purchased from Merck, Darmstadt, Germany. Silica gel 60 (70–230 mesh), Diaion HP-20, and polyamide 6 (Merck, Darmstadt, Germany) were used for different column chromatographic procedures.

### Chemicals

Compounds used for the biological study; 4-Amino-5-methylamino-2′,7′-difluorofluorescein diacetate (DAF-FM) and 2′,7′-dichlorofluorescein diacetate (DCF) were obtained from Molecular Probes, New York, USA. In addition, methylene blue (MB), Nω-Nitro-L-arginine methyl ester hydrochloride (L-NAME), indomethacin (INDO), tetraethylammonium chloride (TEA), and phenylephrine (PE) were obtained from Sigma-Aldrich (Munich, Germany). Ultrapure deionized water was used for dissolving all chemicals except DAF-FM, DCF, and INDO, which were dissolved in dimethylsulphoxide (DMSO). Final DMSO concentration in the assay media did not exceed 0.1 %.

### Plant material

*G. mangostana* fruits were obtained from the market in Kingdom Saudi Arabia in December 2014. The fruits were air dried and a voucher specimen was kept in the herbarium of Faculty of Pharmacy, King Abdulaziz University (no. GM1424). The identity of the plant was kindly authenticated by Dr. Emad Al-Sharif, Associate Professor of Plant Ecology, Dept. of Biology, Faculty of Science & Arts, Khulais, King Abdulaziz University, Saudi Arabia.

### Extraction and isolation

The dried pulverised *G. mangostana* fruit hulls (500 gm) were exhaustively extracted with methanol using Ultraturrax. The collected methanol extracts were evaporated under vacuum to produce a brown residue of the total methanol extract (GMT, 20 g). GMT was suspended in water (500 mL) and fractionated with chloroform to produce a CHCl_3_- soluble fraction (Fr I). The remaining aqeous layer was concentrated and applied to a Diaion HP-20 column (6 × 110 cm, 250 g) and eluted successively with H_2_O, 50 % MeOH and 100 % MeOH (1 L, each). The collected fractions were separately evaporated under vaccum to obtain fractions II (2 gm), III (7 gm) and IV (4 gm), respectively [13]. Part of fraction III (FR. III) was chromatographed on polyamide column (6 × 100 cm, 250 g) and eluted with H_2_O and increasing amounts of MeOH until pure MeOH, the obtained fractions were pooled into four main subfractions (A-D) based on TLC investigations. Subfraction A (10–20 % MeOH) was purified on a silica gel column (25 × 2 cm, 50 g) using CHCl_3_:MeOH (9.5:0.5, *v/v*) to give compounds **1** (14 mg) and **2** (125 mg). Subfraction B (30 % MeOH) was chromatographed on silica gel column (25 × 2 cm, 50 g) and eluted with CHCl_3_:MeOH (9.5:0.5, *v/v*) to afford compounds **3** (32 mg) and **4** (50 mg). Subfraction C (40–60 % MeOH) was applied to silica gel column (25 × 2 cm, 50 g) using CHCl_3_:MeOH (9:1, *v/v*) to yield **5** (150 mg). Finally, Silica gel column (25 × 2 cm, 50 g) of subfraction D (70–100 % MeOH) using CHCl_3_:MeOH (8:2, *v/v*) gave **6** (30 mg).

### Animals

The current study was conducted using 72 male Wistar rats (6–8 weeks old). They were supplied by the Animal house, King Abdulaziz University, Jeddah, Saudi Arabia. Animals were acclimatized in animal facility for seven days before the experiment. They were maintained on a 12-h light–dark cycle, and stable temperature (22 ± 2 oC). Experimental protocol was ethically approved by the Unit of Biomedical Ethics, Faculty of Medicine, King Abdulaziz University (Reference # 329–16).

The metabolic syndrome (MetS) was induced in rats by adding fructose (10 %) to every day drinking water and salt (3 %) to the diet for 12 weeks while control rats received standard diet. The induction of MetS was confirmed by a stable hyperinsulinemia (2.5–3.5 ng/dL) after 12 weeks of high fructose/high salt diet. This protocol was found effective in inducing vascular complications associated with MetS as indicated in previous work from our laboratories [16]. Animals were killed by decapitation and the thoracic aorta was carefully excised and washed with ice-cold Krebs-Henseleit buffer (KHB; NaCl 118.1, KCl 4.69, KH_2_PO_4_ 1.2, NaHCO_3_ 25.0, glucose 11.7, MgSO_4_ 0.5, and CaCl_2_ 2.5 mM). The aorta was cut into rings (~3 mm length) after cleaning from fat and connective tissue. A glucose meter (ACCU-CHEK, Roche, Mannheim, Germany) was used to measure glucose level from tail blood. An immunosorbent assay (ELISA, Millipore, Billerica, MA, USA) with anti-rat insulin monoclonal antibodies was used to measure serum insulin.

### Vascular reactivity

Vascular reactivity of the isolated aortae was performed using the isolated artery techniques as previously described [17–20]. In brief, aortae isolated from MetS animals were incubated with the vehicle (0.1 % DMSO) or different concentrations of GMT (10–100 μg/mL), fractions (I, III, IV) (1–10 μg/mL) or isolated compounds (all at 10–100 μM) for 30 min before studying the vasoconstriction responses to the standard vasoconstrictor phenylephrine (PE). For studying the contractile responsiveness of aortae, increments in tension to cumulative additions of PE (10^−9^ to 10^−5^ M) were recorded.

### Direct vasodilatation

A set of experiments were carried out for investigating the direct vasodilation effect of GMT, fractions (I, III, and IV), or isolated compounds **1**–**6**. In these experiments, cumulative concentrations of GMT or fractions (I, III, and IV) all at concentrations (1–100 μg/mL) or isolated compounds **1**–**6** (Conc. 10–100 μM) were added to the organ bath, containing isolated aortae pre-contracted with PE (10 μM) and the decreases in tension was recorded. Final vehicle concentration (0.4 % DMSO) did not show any effect on PE (10 μM) precontraction in our preliminary data. In other sets of experiments, L-NAME (100 μM), MB (5 μM), TEA (10 mM), INDO (5 μM) or the vehicle (0.1 % DMSO) were added 30 min before investigating the direct vasodilator effect of Fr. III and compounds **2**, **4**, and **5** as above.

### NO generation

The NO generation from isolated aorta was investigated using the fluorescence probes 4-amino-5-methylamino-2′,7′-difluorofluorescein diacetate (DAF-FM) as in previous work from our laboratories [9, 21–23] with some modifications. Briefly, isolated aortic rings (~3 mm length) was added to 96 well black plate wells containing 110 μL saline and 2.5 μM DAF-FM with or without acetylcholine (100 μM), Fr. III (1 μg/mL) or compounds **2**, **4**, and **5** (Conc. 100 μM) for 3 min (incubated at 37 °C). At the end, 90 μL volumes were transferred into new wells and the fluorescence intensity (λex = 485, λex = 525) were measured using monochromator SpectraMax® M3 plate reader (Molecular devices, California, USA). The fluorescence intensity of the DAF-FM plus acetylcholine, Fr. III or any of the compounds before addition of aortic rings were recorded and subtracted to avoid any interference from the tested substance own fluorescence.

### ROS generation

The reactive oxygen species (ROS) generation from isolated aortic rings (~3 mm length) was investigated using the fluorescence probes 2′,7′-dichlorofluorescein diacetate (DCF) as previously described [24, 25] with some modifications. Briefly, isolated aortae were added to 96 well black plate wells, containing 110 μL volumes of saline and 2.5 μM DCF with or without Fr. III (1 μg/mL) or compounds **2**, **4**, and **5** (Conc. 100 μM) for 30 min at 37 °C. At the end 90 μL volumes were transferred to new wells and the fluorescence intensity were measured (λex = 485, λex = 525) using monochromator SpectraMax® M3 plate reader. The fluorescence intensity of the DCF plus tested substances before addition of aortic rings were recorded and subtracted to avoid any interference from the tested substance own fluorescence.

### Statistical analysis

Values are expressed as mean ± SEM and *N* = 6. Statistical analysis was carried out by the one or two (as indicated in figure legends) way analysis of variance (ANOVA) followed by Dunnett’s post hoc test using the statistical software Prism 5® (Graphpad, CA, USA). Probability levels less than 0.05 were considered statistically significant.

## Results

### Metabolic syndrome characteristics

Addition of fructose (10 %) to every day drinking water and salt (3 %) to the diet for 12 weeks led to a significant increase in body weight, fasting blood glucose, insulin and mean arterial blood pressure compared with control rats (Table 1).

**Table 1:** Effect of adding fructose (10 %) to every day drinking water and salt (3 %) to the diet for 12 weeks on the increase in body weight, blood glucose, serum insulin and mean arterial blood pressure (Mean BP) in rats

Treatment	Body weight increase (%)	blood glucose (mg/dl)	Serum insulin (ng/dl)	Mean BP (mmHg)
Control	32.46 ± 5.5	74.4 ± 3.1	0.72 ± 0.08	120.8 ± 1.7
MetS	128.6 ± 33.7^*^	112.8 ± 5.3^*^	2.79 ± 0.41^*^	145.0 ± 2.2^*^

Values are expressed as the mean ± SEM; *N* = 8 animals; ^*^ *P* < 0.05, compared with the corresponding control group values using Unpaired *t* test

### Effect of GMT and fractions

#### Effect on exaggerated vasoconstriction

**Fig. 1:**
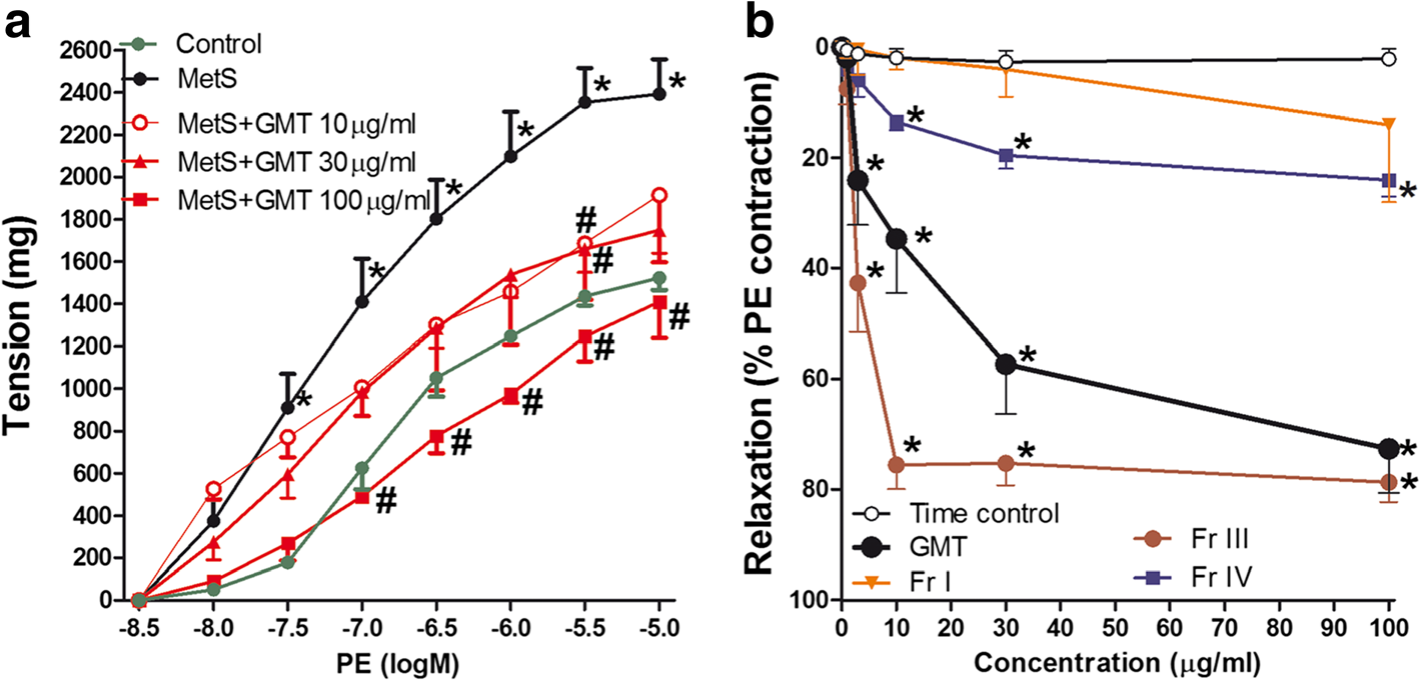
Effect of thirty minutes incubation of different concentrations (10–100 μg/ml) of the mangosten total extract (GMT) on the responsiveness to phenylephrine of aortae isolated from fructose and salt- induced metabolic syndrome (MetS, for 12 weeks). *P < 0.05, compared with the corresponding control values; #*P* < 0.05, compared with the corresponding MetS values; by two Way ANOVA and Dunnett’s post hoc test”. (**a**) and: Effect of cumulative addition (1–100 μg/ml) of GMT and its fractions on phenylephrine pre-contracted MetS aortae. *P < 0.05, compared with the corresponding time control values; by Tow Way ANOVA and Dunnett’s post hoc test”. (**b**). ^*^ *P* < 0.05, compared with the corresponding control values; ^#^ *P* < 0.05, compared with the corresponding MetS values; by Tow Way ANOVA and Dunnett’s *post hoc* test

Aortae isolated from MetS animals showed exaggerated vasoconstriction responses to PE compared to control group (Fig. 1a). Incubation of aortae isolated from MetS animals for 30 min with GMT at final concentrations of 10, 30, and 100 μg/mL significantly alleviated the exaggerated vasoconstriction in a concentration dependent manner (Fig. 1a).

### Direct vasodilation

In search for the bioactive fraction, Fig. 1b showed that the addition of cumulative concentrations (1–100 μg/mL) of GMT as well as fractions (I, III, and IV) to the organ bath led to a concentration dependent vasodilation of isolated aortae pre-contracted with PE (10 μM). Fr. III possessed the main active compounds as it produced the strongest relaxation of PE pre-contracted aortae (Fig. 1b).

### Effect of Fr. III and isolated compounds

#### Phytochemical investigation

**Fig. 2:**
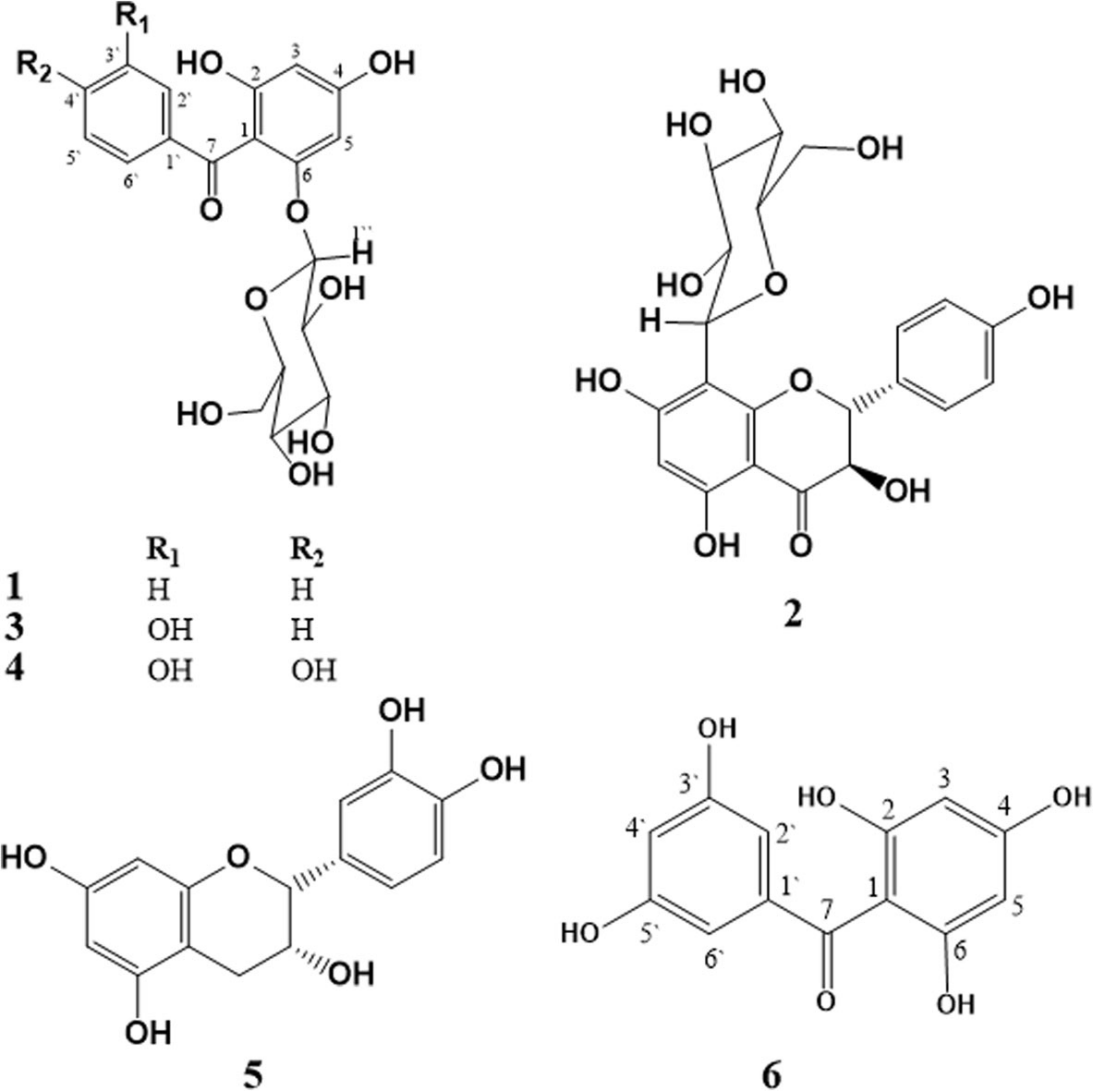
Chemical structures for compounds isolated from *G. mangostana*

Bio-guided fractionation revealed a high bioactivity of Fr. III. While, Fr. II was found to be free sugars and exhibited no polyphenolic characters, by tracing on TLC and PC using AlCl_3_ and FeCl_3_ as well as *p*-anisaldehyde:H_2_SO_4_ spray reagents. Phytochemical investigation of Fr. III resulted in the isolation of six major metabolites (Fig. 2). The structures of isolated compounds were identified based on comparison of their spectral data (1D and 2D NMR) with those previously published and confirmed through co-chromatography with authentic samples as garcimangosone D (**1**) [26], aromadendrin-8-C-*β*-D-glucopyranoside (**2**) [27], 2,4,3′-trihydroxy benzophenone-6-O-*β*-D-glucopyranoside (**3**) [28], maclurin-6-O-*β*-D-glucopyranoside (rhodanthenone) (**4**) [28], epicatechin (**5**) [29], and 2,3′,4,5′,6-pentahydroxy benzophenone (**6**) [30] (Additional file 1: Figures S1-S12 and Tables S1 & S2).

### Effect on exaggerated vasoconstriction

**Fig. 3:**
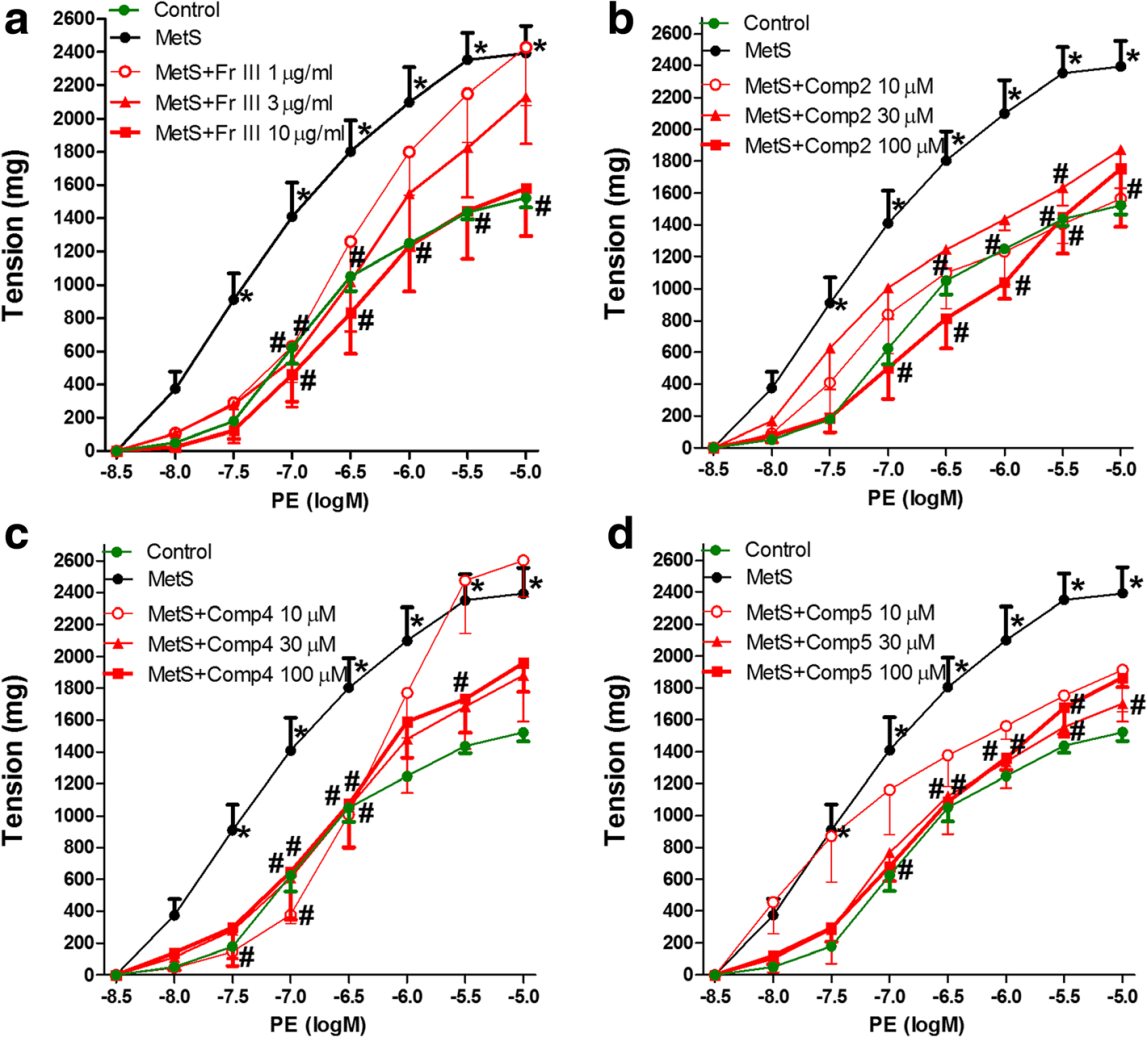
Effect of 30 min in vitro incubation with Fr. III (**a**) and compounds **2 (b)**, **4 (c) **, and **5 (d) ** on the responsiveness to PE of aortae isolated from fructose and salt-induced metabolic syndrome (MetS, for 12 weeks). ^*^ *P* < 0.05, compared with the corresponding control values; ^#^ *P* < 0.05, compared with the corresponding MetS values; by Tow Way ANOVA and Dunnett’s *post hoc* test

The responsibility of Fr. III and compounds **2**, **4**, and **5 to alleviate** exaggerated vasoconstriction produced by the total extract was confirmed. Figure 3 showed that incubation with only Fr. III (1, 3, and 10 μg/mL) alleviated the exaggerated vasoconstriction of MetS aortae (Fig. 3a). Similar alleviations of MetS aortae exaggerated response were observed after 30 min incubation with 10, 30, and 100 μM of **2** (Fig. 3b), **4** (Fig. 3c), and **5** (Fig. 3d), respectively.

### Direct vasodilation

**Fig. 4:**
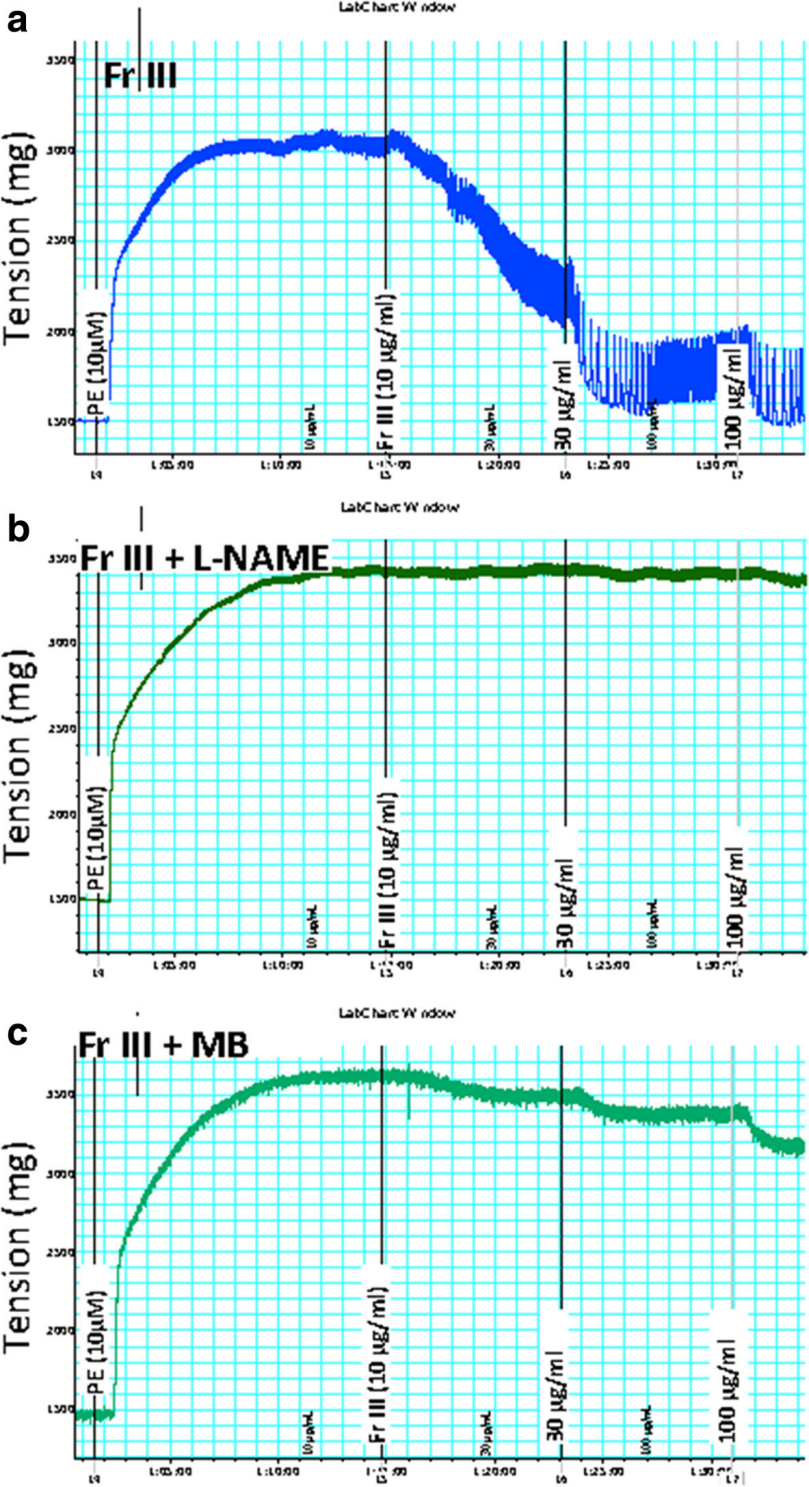
Representative charts for the effect of cumulative addition of Fr. III (1–10 μg/mL) in absence (**a**) or presence of Nω-Nitro-L-arginine methyl ester hydrochloride (L-NAME, 100 μM, **b**) or, methylene blue (MB, 5 μM, **c**) on phenylephrine (PE 10 μM) pre-contracted aortae isolated from normal Wistar rats. L-NAME, MB or the vehicle were added 30 min before investigating the vasodilator effect

**Fig. 5:**
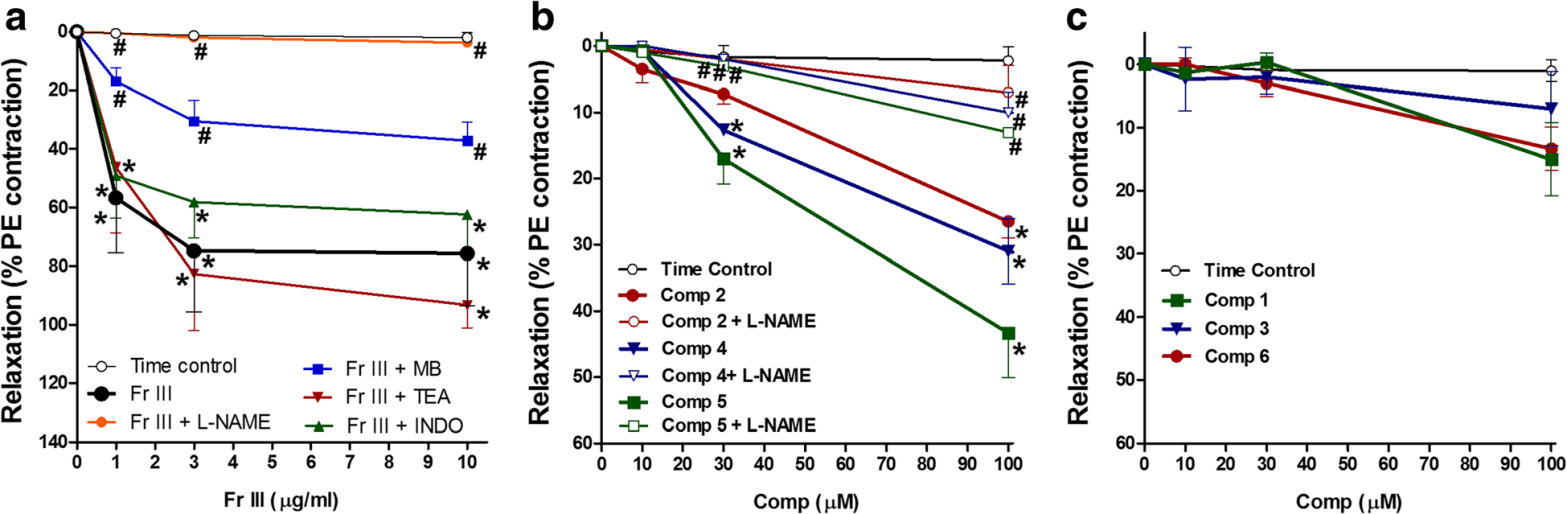
The effect of cumulative addition of Fr. III (1–10 μg/mL, **a**) and compounds (**1**–**6**, 10–100 μM, **b** and **c**) on phenylephrine (PE 10 μM) pre-contracted isolated aortae. Nω-Nitro-L-arginine methyl ester hydrochloride (L-NAME, 100 μM), methylene blue (MB, 5 μM), tetraethylammonium chloride (TEA, 10 mM) and indomethacin (INDO, 5 μM) or the vehicle (0.1 % DMSO) were added 30 min before investigating the vasodilator effect. ^*^ *P* < 0.05, compared with the corresponding control values; ^#^ *P* < 0.05, compared with the corresponding compound values; by Tow Way ANOVA and Dunnett’s *post hoc* test

Addition of cumulative concentrations of Fr. III (1–10 μg/mL) to the organ bath led to a concentration dependent vasodilation of PE pre-contracted aortae (Figs. 4a and 5a). Similarly, addition of cumulative concentrations of compounds **2**, **4**, and **5** (Conc. 10–100 μM) to the organ bath led to a concentration dependent vasodilation (Fig. 5b). However, **1**, **3**, and **6** had no significant vasodilation (Fig. 5c).

Thirty minutes pre-incubation with the nitric oxide synthase inhibitor Nω-nitro-L-arginine methyl ester hydrochloride (L-NAME, 100 μM) before the cumulative addition of Fr. III completely blocked Fr. III vasodilation (Figs. 4b and 5a). Pre-incubation with the guanylate cyclase inhibitor methylene blue (MB, 5 μM), partially inhibited the produced vasodilation (Figs. 4c and 5a). While, pre-incubation with the calcium-activated potassium channels blocker tetraethylammonium chloride (TEA, 10 mM), or the cyclooxygenase inhibitor indomethacin (INDO, 5 μM) did not significantly affect Fr. III-induced vasodilation (Fig. 5a). Similarly, thirty minutes pre-incubation with L-NAME significantly inhibited the produced vasodilation of compounds **2**, **4**, and **5** (Fig. 5b).

### Nitric oxide generation

**Fig. 6:**
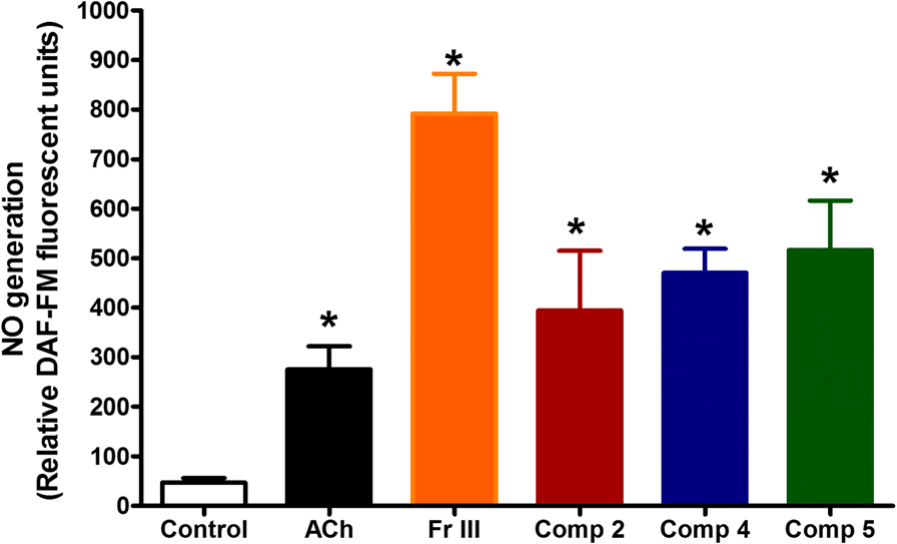
The nitric oxide generating effect of Fr III (10 μg/mL) and compounds (**2**, **4** & **5** at conc. 100 μM)) in aortae isolated from normal animals *P < 0.05, compared with the corresponding control values; by One Way ANOVA and Dunnett's Multiple Comparison Test”

Inserting isolated aortae in media, containing Fr. III (10 μg/mL) produced significant NO generation compared with control. Similarly, compounds **2**, **4**, and **5** (Conc. 100 μM) significantly generated NO from isolated aortae compared with control to a level similar to acetylcholine (Fig. 6).

### Effect on ROS generation

**Fig. 7:**
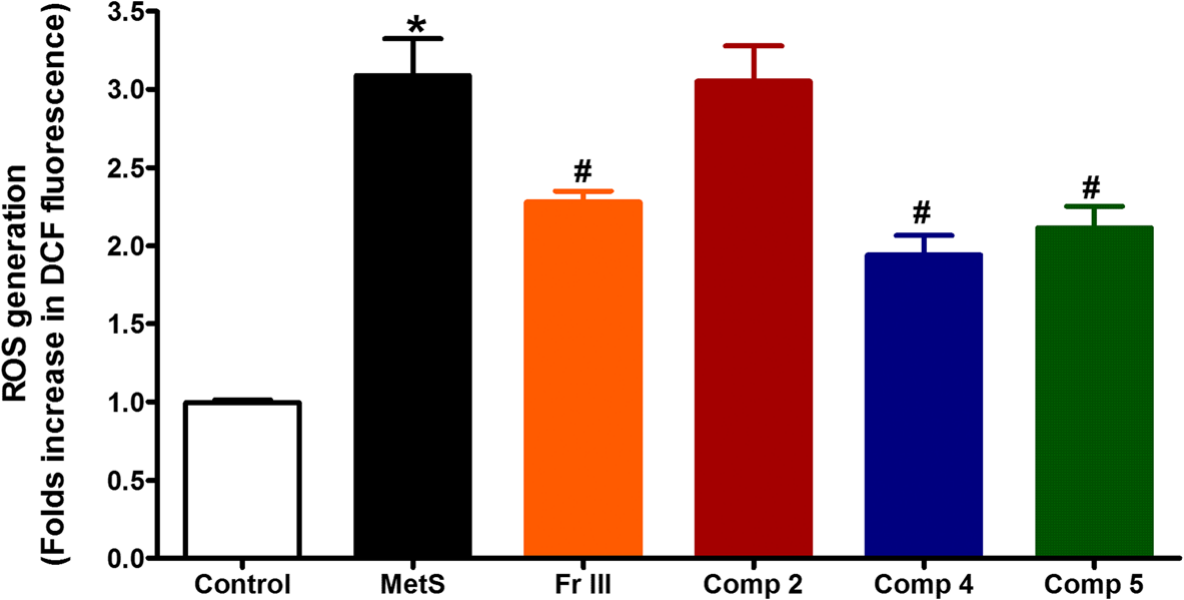
Effect of 30 min in vitro incubation with Fr. III and compounds **2**, **4**, and **5** on ROS production by aortae isolated from fructose-induced metabolic syndrome (MetS, 10 % in drinking water, for 12 weeks). ^*^ *P* < 0.05, compared with the corresponding control values; ^#^ *P* < 0.05, compared with the corresponding MetS group values; by One Way ANOVA and Dunnett’s Multiple Comparison Test

Overproduction of ROS was observed from aortae isolated from MetS animals compared to control. Thirty minutes pre-incubation with Fr. III (10 μg/mL) significantly inhibited ROS generation from MetS aortae (*p* < 0.01). Similarly, compounds **4** and **5** (Conc. 100 μM) significantly inhibited ROS generation from MetS aortae. However, compound **2** did not affect ROS generation from MetS aortae (Fig. 7).

## Discussion

The current study is the first report on the protective effect of *G. mangostana* (GM) on exaggerated vasoconstriction in MetS. A multidisciplinary approach was employed for studying vasoconstriction, dilatation, NO, and ROS production to find out the active metabolite in GM through bioassay-guided process.

It is widely accepted that development of hypertension in normal persons and in MetS is dependent greatly on changes in vascular reactivity. Exaggerated vasoconstriction and or impaired vasodilation are usually correlated with increase in blood pressure [31, 32]. In the current study, isolated aortae from MetS animals showed overstated response upon exposure to PE which is in agreement with previous reports showing that different vasoconstrictors produced exaggerated response in MetS [33, 34]. Several reports revealed the role of phenolic compounds in alleviating the exaggerated vasoconstriction and hence alleviating hypertension in MetS [8, 9]. GM is known for its high content of phenolic constituents such as xanhtone, flavonoids and benzophenones and its anti-diabetic activity [15].

In the present study, GMT significantly alleviated the exaggerated vasoconstriction in MetS aortae in a concentration dependent manner. This effect seemed to be mediated by direct vasodilatation activity as indicated by the data presented here. The bioassay-guided fractionation procedures revealed that Fr. III was mainly responsible for the observed activity while fraction I (xanthone rich fraction) showed weak activity. Chemical investigation of the bioactive fraction resulted in isolation of six major metabolites (**1**–**6**). Only compounds **2**, **4**, and **5** showed significant vasodilation suggesting that they are the main active metabolites responsible for the observed vasodilation in Fr. III and hence the total extract of GM.

The observed vasodilation produced by Fr III as well as compounds **2**, **4**, and **5** seemed to be through stimulating NO generation from the vasculature as Fr. III relaxation was completely blocked by the nitric oxide synthase inhibitor and partially inhibited by guanylate cyclase inhibitor. Meanwhile, it was not affected by the calcium-activated potassium channels blocker or the cyclooxygenase inhibitor. The vasorelaxation responses of Fr. III are almost similar to the responses of compounds **2**, **4**, and **5**. The reason may be competition between the active compounds on the same relaxation pathway as they are all inhibited by L-NAME and MB as have not found any vasoconstricting compound among the isolated metabolites of Fr. III. This suggests stimulating NO generation as a main mechanism by which Fr III as well as compounds **2**, **4**, and 5 induce vasodilation. The NO generation measurement in the present work reinforced this assumption as Fr. III generated nitric oxide from isolated vessels even better than acetylcholine (standard endothelial NO generator). The same was true for vasorelaxation activity. In addition, compounds **2**, **4**, and **5** generated nitric oxide to a level comparable to acetylcholine. The ability of flavonoid nucleus as in **2** to stimulate NO generation was previously reported [9], meanwhile; benzophenone nucleus in **4** is reported here for the first time for this activity. Furthermore, epicatechin (**5**) (flavanol nucleus) was known for its vasorelaxant activity through increasing NO levels in the vasculature [35, 36]. It was reported that the NO-preserving activity of (−)-epicatechin on vascular endothelial cells was due to inhibition of endothelial NADPH oxidase activity [37].

The assumption that compounds **2**, **4**, and **5** are the main active metabolites responsible for activity of Fr. III and hence the total extract of GM was confirmed by the observed alleviation of exaggerated vasoconstriction when incubating them with MetS aortae for only thirty minutes.

The involvement of ROS (endogenous or exogenous) in vascular tone by acting as a mediator for signal transduction in endothelial cells was reported before [34, 38, 39]. In this work, fructose was used to induce MetS resulting in elevation of ROS as previously reported [40, 41]. Production of ROS in MetS resulted in occurrence of different vascular diseases which could be attributed to the ability of ROS to decrease bioavailability of NO and endothelial dysfunction. Moreover, ROS is responsible for vascular diseases through its ability to produce many oxidized and nitrated secondary metabolites [42]. In the present study, Fr. III and compounds **4** and **5** suppressed ROS in MetS aortae. This could also be one of the mechanisms by which GM alleviated exaggerated vasoconstriction and induced vasodilation through increasing NO bioavailability as a result of inhibiting ROS.

## Conclusion

GM significantly inhibited the exaggerated vasoconstriction in MetS. Fr. III and isolated compounds **2**, **4**, and **5** are the most effective ones from the extracts who are responsible for the observed activity through vasodilatation-NO generation mechanism.

## Additional file

Additional file 1: Supplementary document **Table S1.** NMR spectral data of compounds 1–3, **Table S2.** NMR spectral data of compounds 4–6. **Figure S1**-**S12**; 1H and 13C NMR data of compounds 1–6. (DOC 4189 kb)
