# Supplementary material for: Phenolics from Garcinia mangostana alleviate exaggerated vasoconstriction in metabolic syndrome through direct vasodilatation and nitric oxide generation
Source: BMC Complement Altern Med. 2016 Sep 13;16(1):359. doi: 10.1186/s12906-016-1340-5 (PMC5020522; doi:10.1186/s12906-016-1340-5)
Supplement: Additional file 1: — Supplementary document Table S1. NMR spectral data of compounds 1–3, Table S2. NMR spectral data of compounds 4–6. Figure S1-S12; 1H and 13C NMR data of compounds 1–6. (DOC 4189 kb) [file 12906_2016_1340_MOESM1_ESM.doc]

**Additional file 1: Supplementary Materials**

**Phenolics from *Garcinia mangostana* alleviate exaggerated vasoconstriction in metabolic syndrome through direct vasodilatation and nitric oxide generation**

Hossam M. Abdallah^1,2^, Hany El-Bassossy^3,4^, Gamal A. Mohamed^1,5*^, Ali M. El-halawany^1,2^, Khalid Z. Alshali^6^, Zainy M. Banjar^7^

1

*^1^Department of Natural Products, Faculty of Pharmacy, King Abdulaziz University, Jeddah, 21589, Saudi Arabia.*

*^2^Department of Pharmacognosy, Faculty of Pharmacy, Cairo University, Cairo, 11562, Egypt.*

*^3^Department of Pharmacology, Faculty of Pharmacy, King Abdulaziz University, Jeddah, 21589, Saudi Arabia*

*^4^Department of Pharmacology, Faculty of Pharmacy, Zagazig University, Zagazig, Egypt*

*^5^Pharmacognosy Department, Faculty of Pharmacy, Al-Azhar University, Assiut Branch, Assiut 71524, Egypt*

*^6^Departement of Medicine, Faculty of Medicine, King Abdulaziz University, Jeddah, 21589, Saudi Arabia*

*^7^Departement of Clinical Biochemistry, Faculty of Medicine, King Abdulaziz University, Jeddah, 21589, Saudi Arabia*

* Corresponding author at: Department of Natural Products, Faculty of Pharmacy, King Abdulaziz University, Jeddah, 21589, Saudi Arabia. Tel .: +966 597636182. E-mail address: [gamals2001@yahoo.com](mailto:gamals2001@yahoo.com)

List of supplementary materials

|  | **Page** |
| --- | --- |
| **Table S1. NMR spectral data of compounds 1-3 (CD_3_OD, 400 and 100 MHz).** | **3** |
| **Table S2. NMR spectral data of compounds 4-6 (CD_3_OD, 400 and 100 MHz).** | **4** |
| **Fig. S1.** ^1^H NMR spectrum of compound **1** (CD_3_OD, 400 MHz). | **5** |
| **Fig. S2.** ^13^C NMR spectrum of compound **1** (CD_3_OD, 100 MHz). | **5** |
| **Fig. S3.** ^1^H NMR spectrum of compound **2** (CD_3_OD, 400 MHz). | **6** |
| **Fig. S4.** ^13^C NMR spectrum of compound **2** (CD_3_OD, 100 MHz). | **6** |
| **Fig. S5.** ^1^H NMR spectrum of compound **3** (CD_3_OD, 400 MHz). | **7** |
| **Fig. S6.** ^13^C NMR spectrum of compound **3** (CD_3_OD, 100 MHz). | **7** |
| **Fig. S7.** ^1^H NMR spectrum of compound **4** (CD_3_OD, 400 MHz). | **8** |
| **Fig. S8.** ^13^C NMR spectrum of compound **4** (CD_3_OD, 100 MHz). | **8** |
| **Fig. S9.** ^1^H NMR spectrum of compound **5** (CD_3_OD, 400 MHz). | **9** |
| **Fig. S10.** ^13^C NMR spectrum of compound **5** (CD_3_OD, 100 MHz). | **9** |
| **Fig. S11.** ^1^H NMR spectrum of compound **6** (CD_3_OD, 400 MHz). | **10** |
| **Fig. S12.** ^13^C NMR spectrum of compound **6** (CD_3_OD, 100 MHz). | **10** |

**Table S1. NMR spectral data of compounds 1-3 (CD_3_OD, 400 and 100 MHz).**

| No | **1** | | **2** | | **3** | |
| --- | --- | --- | --- | --- | --- | --- |
|  | **δ_H_ [mult., *J*(Hz)]** | **δ_C_ (mult.)** | **δ_H_ [mult., *J*(Hz)]** | **δ_C_ (mult.)** | **δ_H_ [mult., *J*(Hz)]** | **δ_C_ (mult.)** |
| 1 | - | 109.1 (C) | - | - | - | 109.4 (C) |
| 2 | - | 162.0 (C) | 4.96 d (12.4) | 84.9 (CH) | - | 161.7 (C) |
| 3 | 6.06 d (2.0) | 98.2 (CH) | 4.52 d (12.4) | 73.6 (CH) | 6.06 d (2.0) | 98.1 (CH) |
| 4 | - | 164.0 (C) | - | 198.9 (C) | - | 163.8 (C) |
| 5 | 6.24 d (2.0) | 95.8 (CH) | - | 163.9 (C) | 6.21 d (2.0) | 95.7 (CH) |
| 6 | - | 159.7 (C) | 5.95 s | 96.5 (CH) | - | 159.5 (C) |
| 7 | - | 199.5 (C) | - | 167.6 (C) | - | 199.3 (C) |
| 8 |  |  | - | 105.6 (C) |  |  |
| 9 | - | - | - | 164.1 (C) | - | - |
| 10 | - | - | - | 101.8 (C) | - | - |
| 1` | - | 141.7 (C) | - | 122.2 (C) | - | 142.8 (C) |
| 2` | 7.69 m | 130.1 (CH) | 7.33 d (8.4) | 130.4 (CH) | 7.11 m | 116.9 (CH) |
| 3` | 7.38 m | 129.0 (CH) | 6.82 d (8.4) | 116.2 (CH) | - | 158.2 (C) |
| 4` | 7.49 m | 133.2 (CH) | - | 159.2 (C) | 6.93 m | 120.4 (CH) |
| 5` | 7.38 m | 129.0 (CH) | 6.82 d (8.4) | 116.2 (CH) | 7.18 m | 130.2 (CH) |
| 6` | 7.69 m | 130.1 (CH) | 7.33 d (8.4) | 130.4 (CH) | 7.16 m | 121.4 (CH) |
| 1`` | 4.85 d (7.8) | 101.7 (CH) | 4.78 d (7.6) | 75.1 (CH_2_) | 4.85 d (7.8) | 101.6 (CH) |
| 2`` | 2.80 m | 74.5 (CH) | 4.12 m | 72.6 (CH) | 2.89 m | 74.6 (CH) |
| 3`` | 3.23 m | 77.8 (CH) | 3.46 m | 80.2 (CH) | 3.32 m | 77.8 (CH) |
| 4`` | 3.18 m | 71.1 (CH) | 3.43 m | 71.9 (CH) | 3.23 m | 70.9 (CH) |
| 5`` | 3.4 m | 78.2 (CH) | 3.30 m | 82.5 (CH) | 3.4 m | 78.2 (CH) |
| 6`` | 3.82 dd (12.0, 2.0)  3.63 dd (12.0, 5.6) | 62.5 (CH_2_) | 3.84 dd (12.0, 2.0)  3.70 dd (12.0, 5.2) | 63.0 (CH_2_) | 3.83 dd (12.0, 2.0)  3.63 dd (12.0, 5.6) | 62.4 (CH_2_) |

**Table S2. NMR spectral data of compounds 4-6 (CD_3_OD, 400 and 100 MHz).**

| No | **4** | | **5** | | **6** | |
| --- | --- | --- | --- | --- | --- | --- |
|  | **δ_H_ [mult., *J*(Hz)]** | **δ_C_ (mult.)** | **δ_H_ [mult., *J*(Hz)]** | **δ_C_ (mult.)** | **δ_H_ [mult., *J*(Hz)]** | **δ_C_ (mult.)** |
| 1 | - | 110.6 (C) | - | - | - | 106.4 (C) |
| 2 | - | 159.5 (C) | 4.82 s | 79.9 (CH) | - | 161.8 (C) |
| 3 | 6.06 d (2.0) | 98.2 (CH) | 4.71 m | 67.5 (CH) | 5.83 s (2.0) | 95.7 (CH) |
| 4 | - | 162.2 (C) | 2.87 dd (16.6, 4.8)  2.73 dd (16.6, 2.8) | 29.3 (CH_2_) | - | 165.5 (C) |
| 5 | 6.24 d (2.0) | 95.8 (CH) | - | 157.4 (C) | 5.83 s (2.0) | 95.7 (CH) |
| 6 | - | 158.6 (C) | 5.91 d (2.4) | 96.4 (CH) | - | 163.4 (C) |
| 7 | - | 197.7 (C) |  | 158.0 (C) | - | 200.6 (C) |
| 8 |  |  | 5.94 d (2.4) | 95.9 (CH) |  |  |
| 9 | - | - | - | 157.7 (C) | - | - |
| 10 | - | - | - | 100.1 (C) | - | - |
| 1` | - | 132.6 (C) | - | 132.3 (C) | - | 145.7 (C) |
| 2` | 7.28 d (2.0) | 117.8 (CH) | 6.97 d (2.0) | 115.4 (CH) | 6.73 s | 106.4 (CH) |
| 3` | - | 145.9 (C) | - | 145.8 (C) | - | 159.2 (C) |
| 4` | - | 152.0 (C) | - | 146.0 (C) | 6.52 m | 107.7 (CH) |
| 5` | 6.76 d (8.4) | 115.6 (CH) | 6.75 d (8.4) | 115.9 (CH) |  | 159.2 (CH) |
| 6` | 7.21 d (8.4) | 124.8 (CH) | 6.80 dd (8.4, 2.0) | 119.4 (CH) | 6.73 s | 106.4 (CH) |
| 1`` | 4.85 d (7.8) | 102.3 (CH) | - | - |  |  |
| 2`` | 3.11dd (9.2, 7.6) | 74.7 (CH) | - | - |  |  |
| 3`` | 3.34 m | 77.8 (CH) | - | - | - | - |
| 4`` | 3.29 m | 71.0 (CH) | - | - | - | - |
| 5`` | 3.35 m | 78.2 (CH) | - | - | - | - |
| 6`` | 3.85 dd (12.0, 2.0)  3.67 dd (12.0, 5.6) | 63.0 (CH_2_) |  |  |  |  |

**
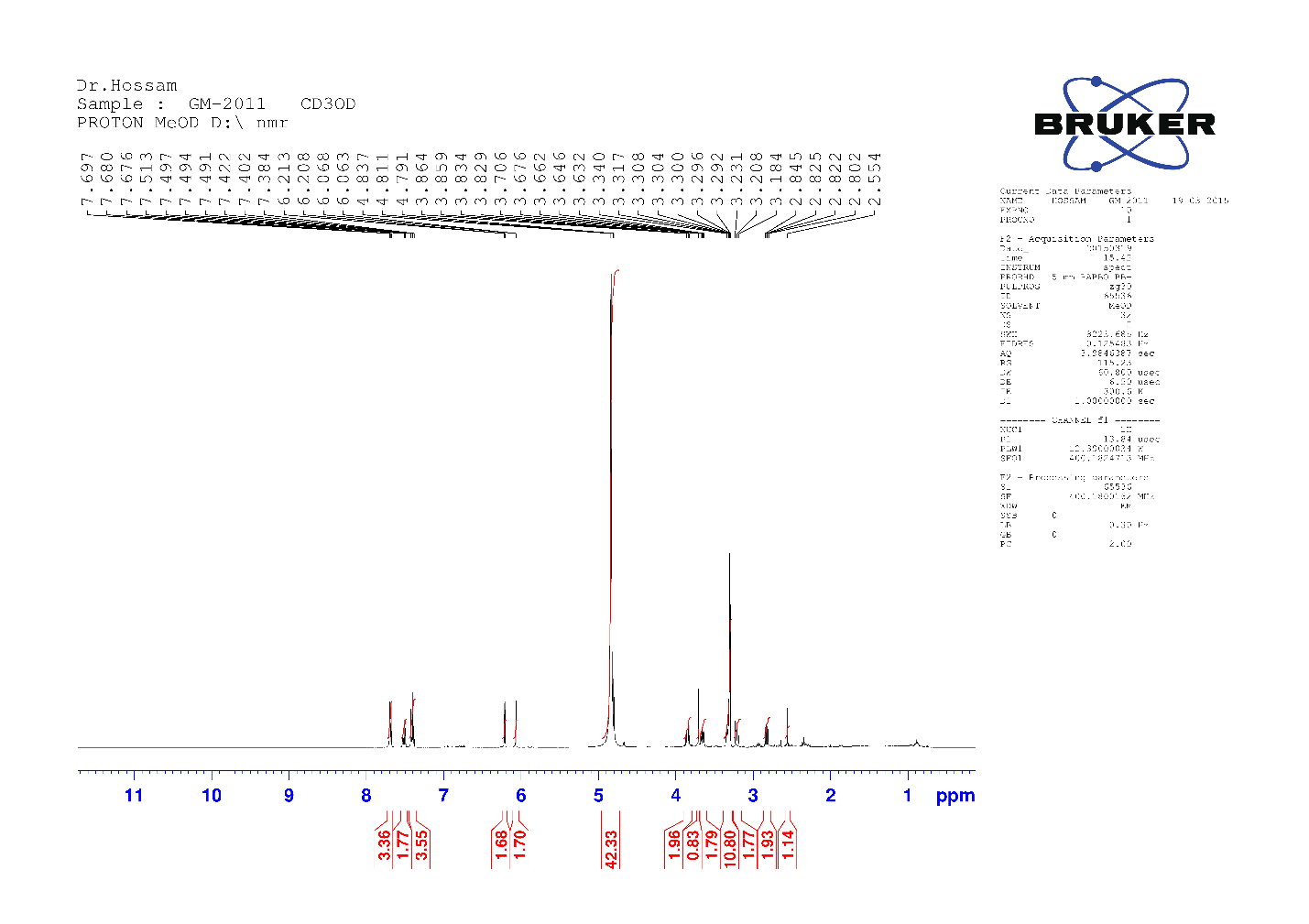
**

**Fig. S1.** ^1^H NMR spectrum of compound **1** (CD_3_OD, 400 MHz).

**
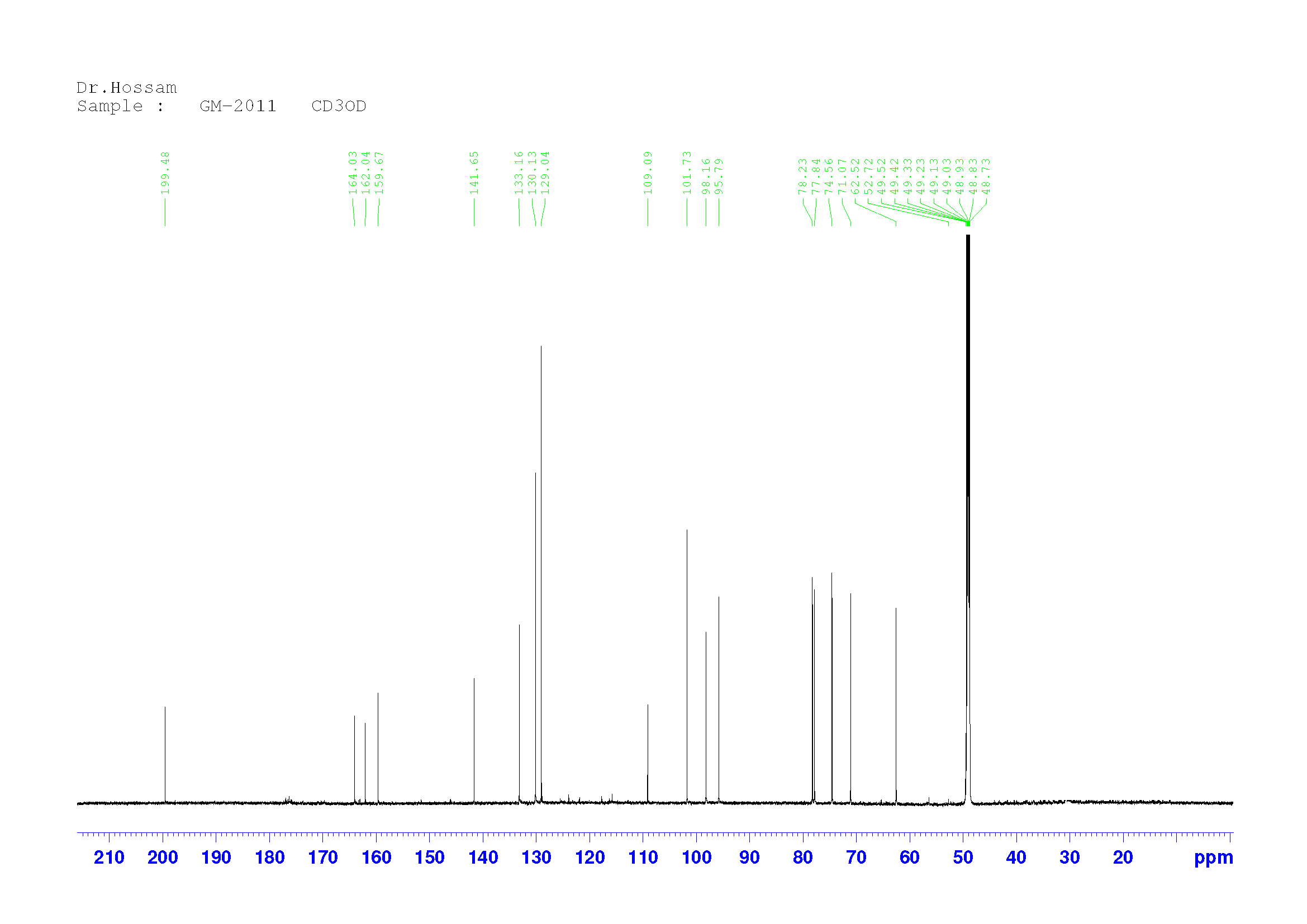
**

**Fig. S2.** ^13^C NMR spectrum of compound **1** (CD_3_OD, 100 MHz).

**
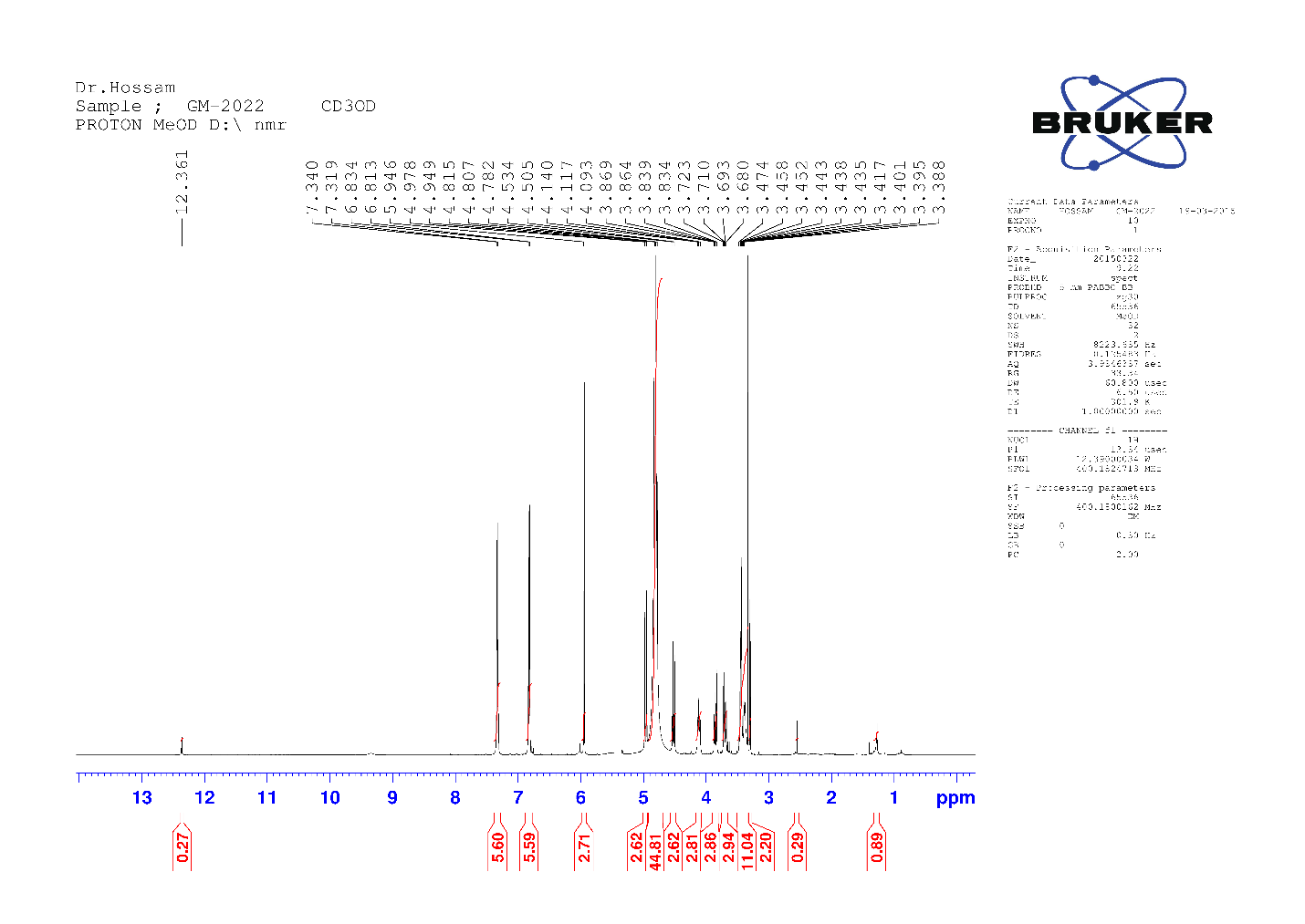
**

**Fig. S3.** ^1^H NMR spectrum of compound **2** (CD_3_OD, 400 MHz).

**
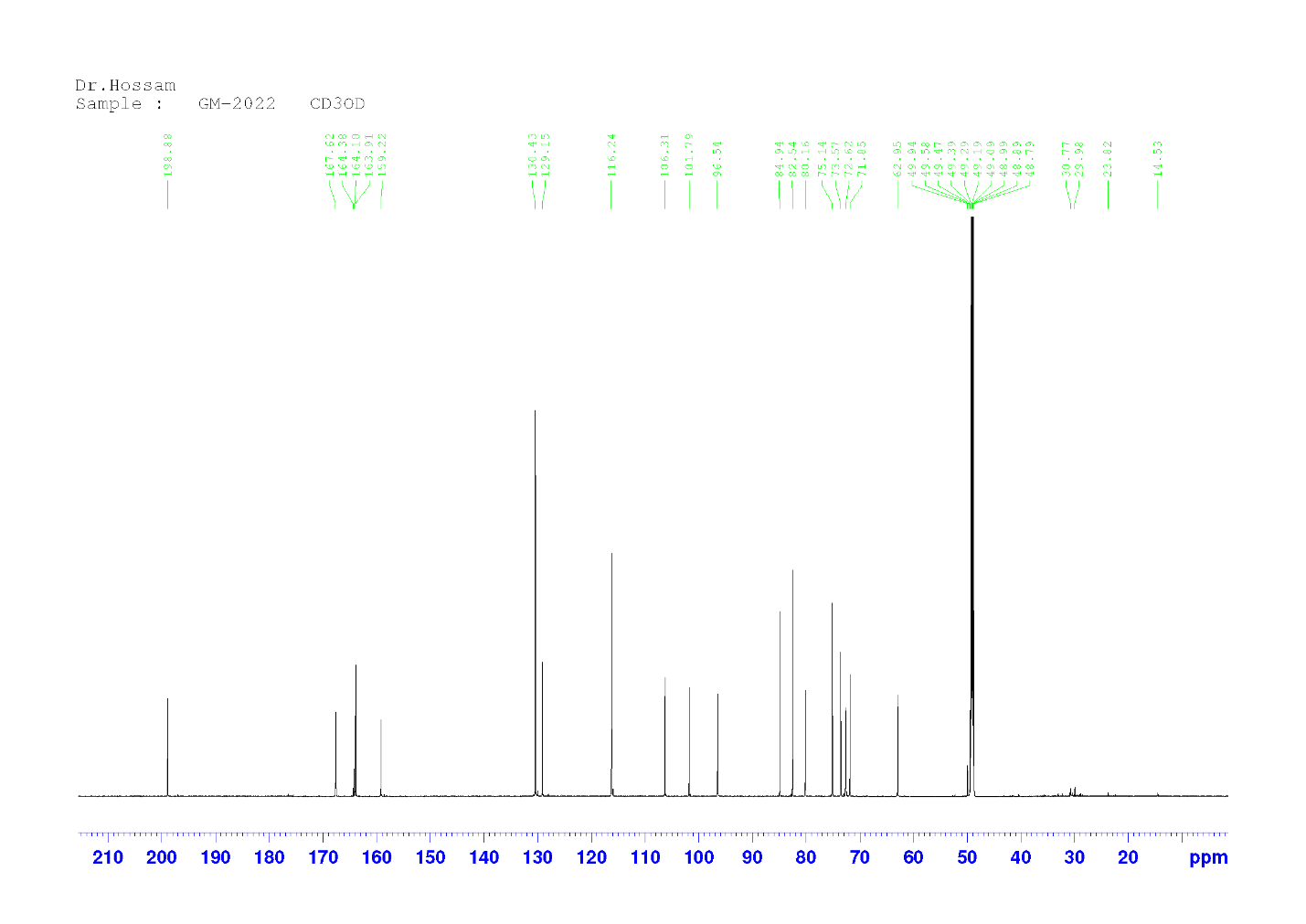
**

**Fig. S4.** ^13^C NMR spectrum of compound **2** (CD_3_OD, 100 MHz).

**Fig. S5.** ^1^H NMR spectrum of compound **3** (CD_3_OD, 400 MHz).

**
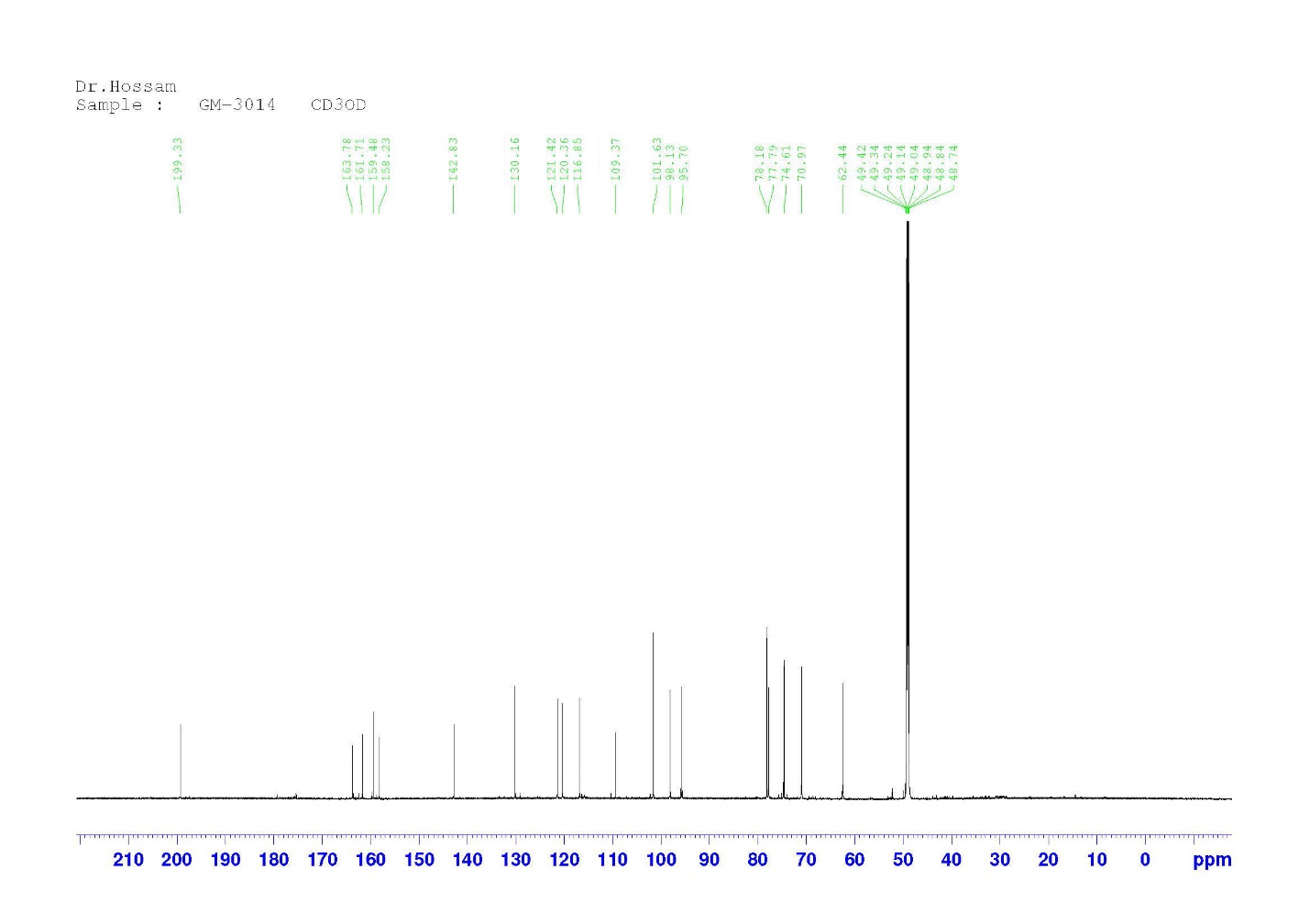
**

**Fig. S6.** ^13^C NMR spectrum of compound **3** (CD_3_OD, 100 MHz).

**Fig. S7.** ^1^H NMR spectrum of compound **4** (CD_3_OD, 400 MHz).

**
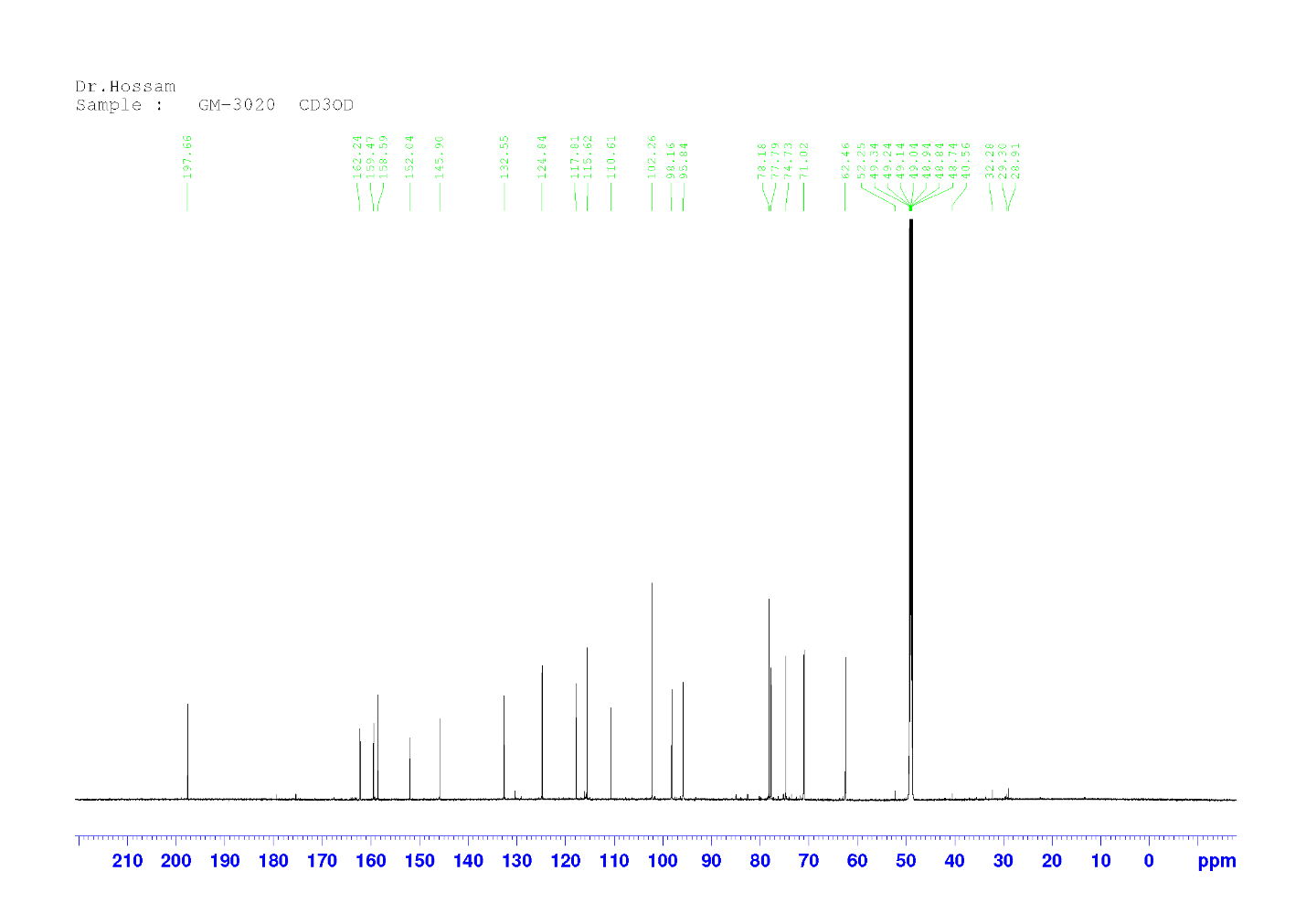
**

**Fig. S8.** ^13^C NMR spectrum of compound **4** (CD_3_OD, 100 MHz).

**
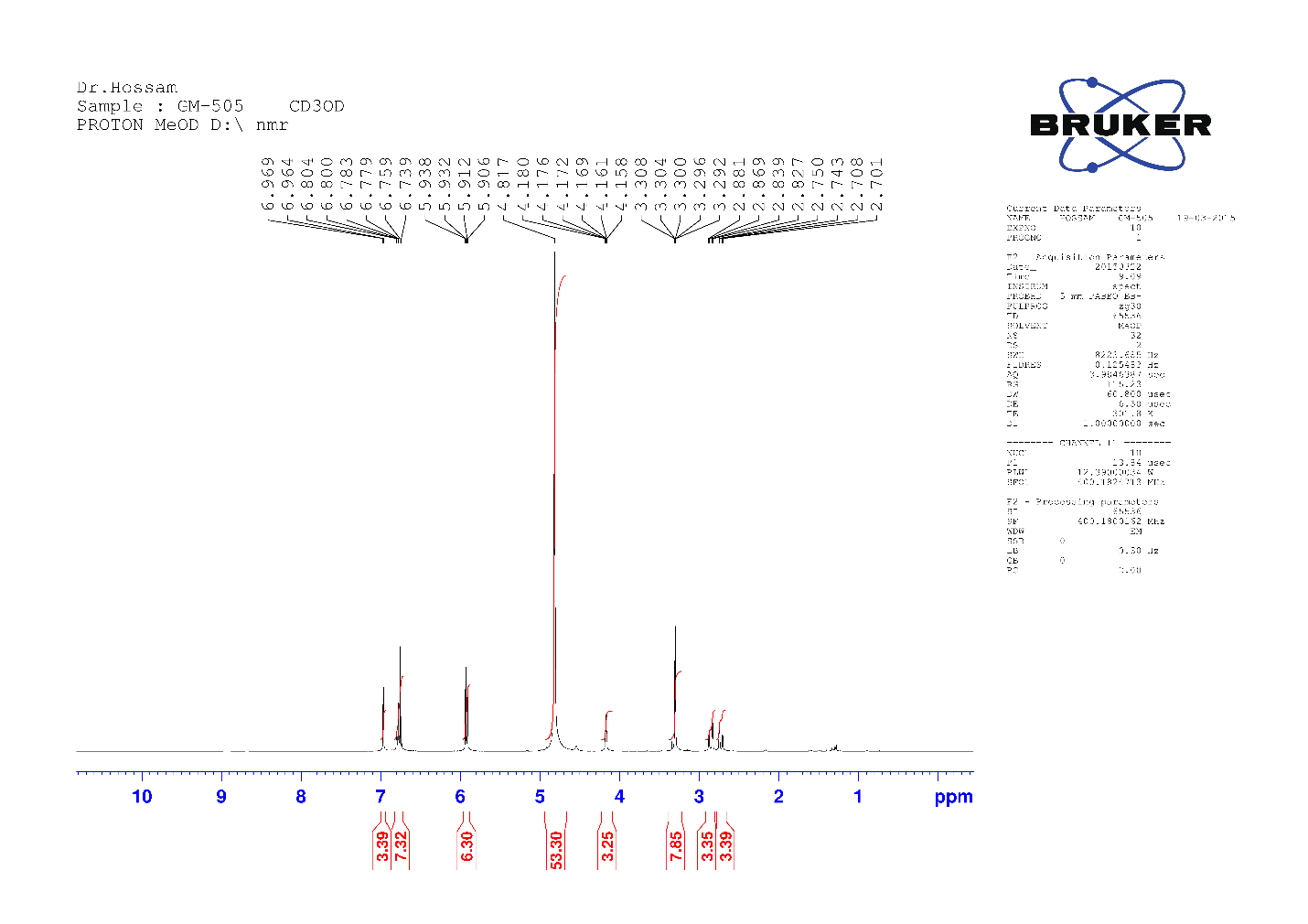
**

**Fig. S9.** ^1^H NMR spectrum of compound **5** (CD_3_OD, 400 MHz).

**
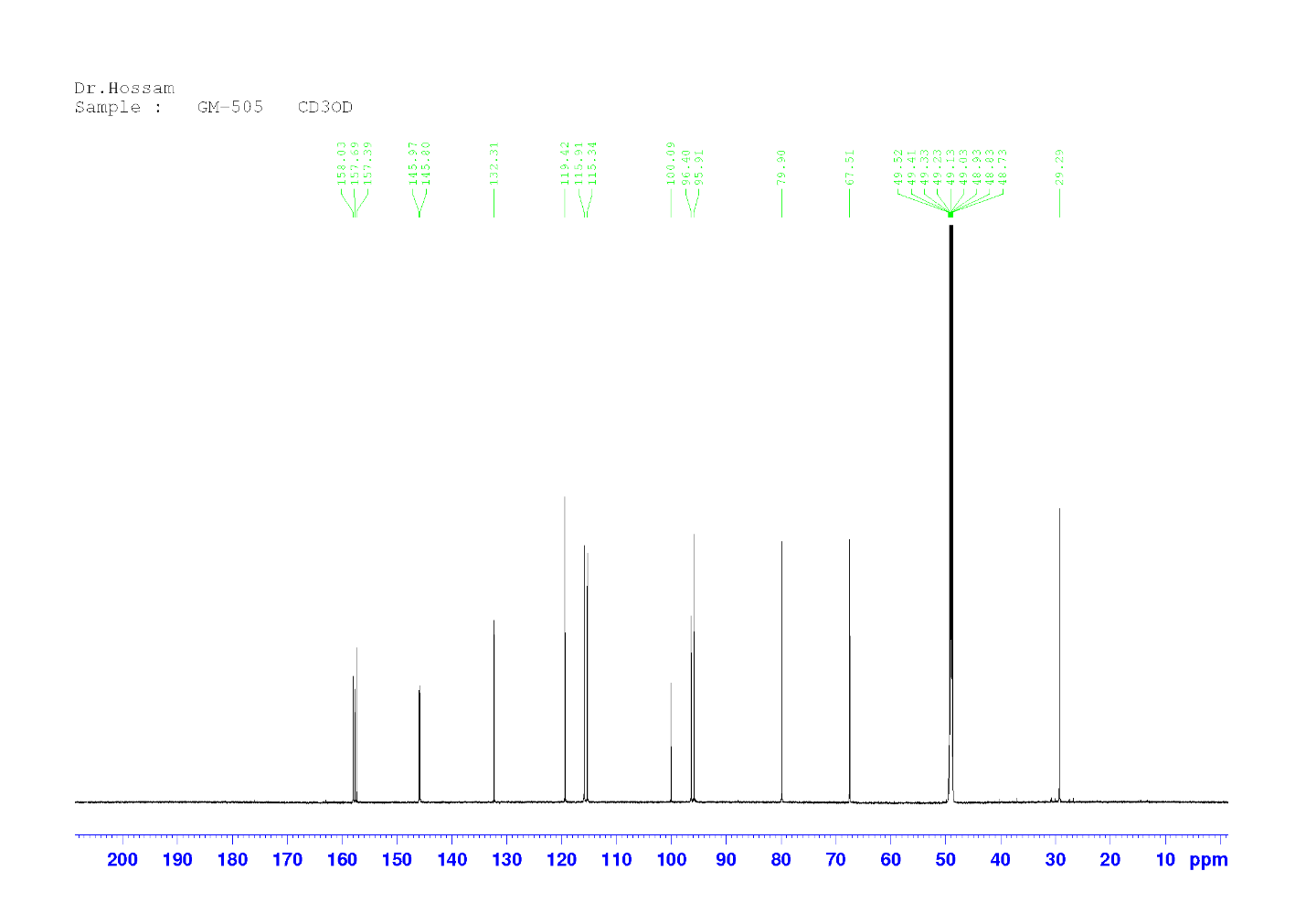
**

**Fig. S10.** ^13^C NMR spectrum of compound **5** (CD_3_OD, 100 MHz).

**
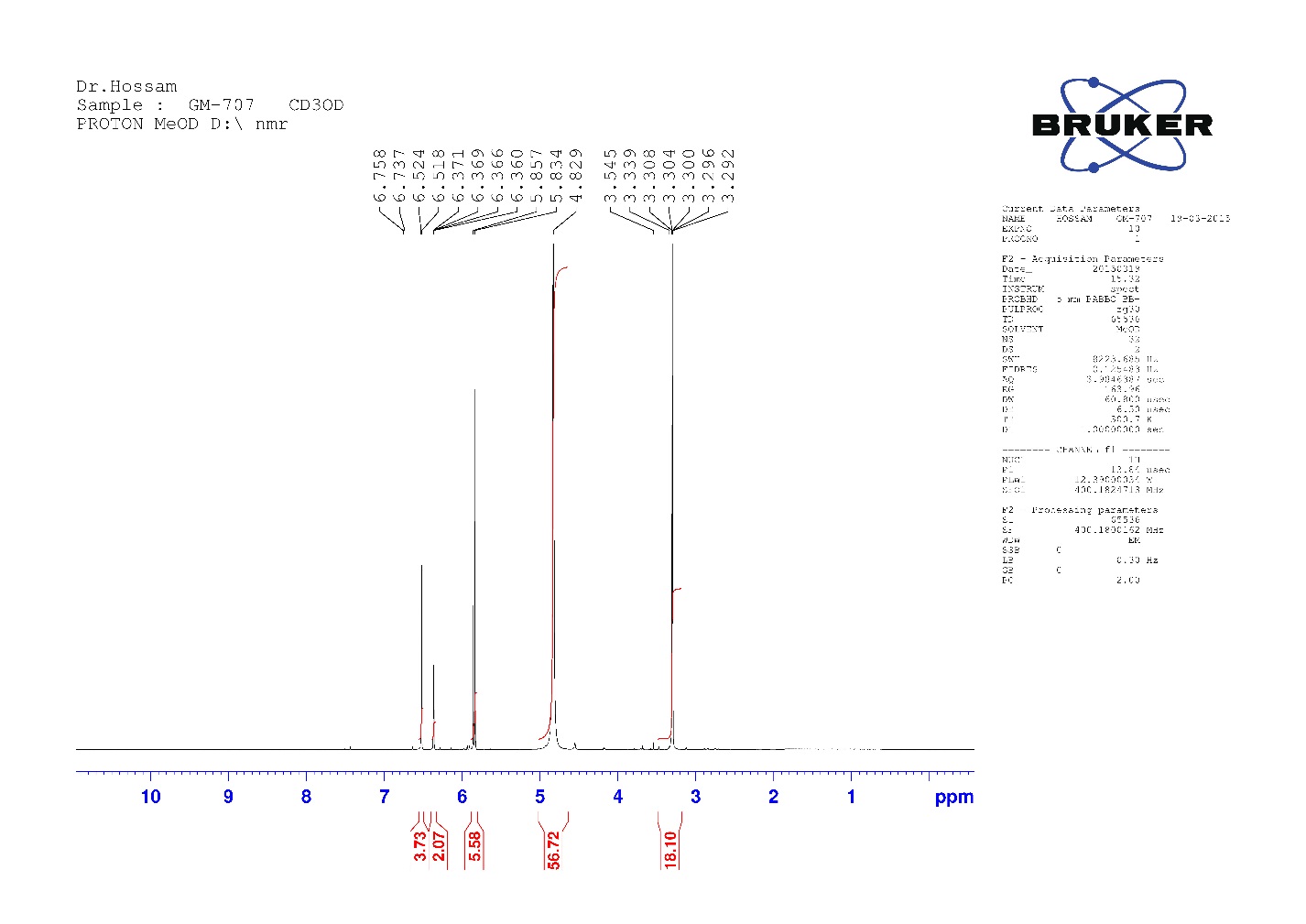
**

**Fig. S11.** ^1^H NMR spectrum of compound **6** (CD_3_OD, 400 MHz).

**
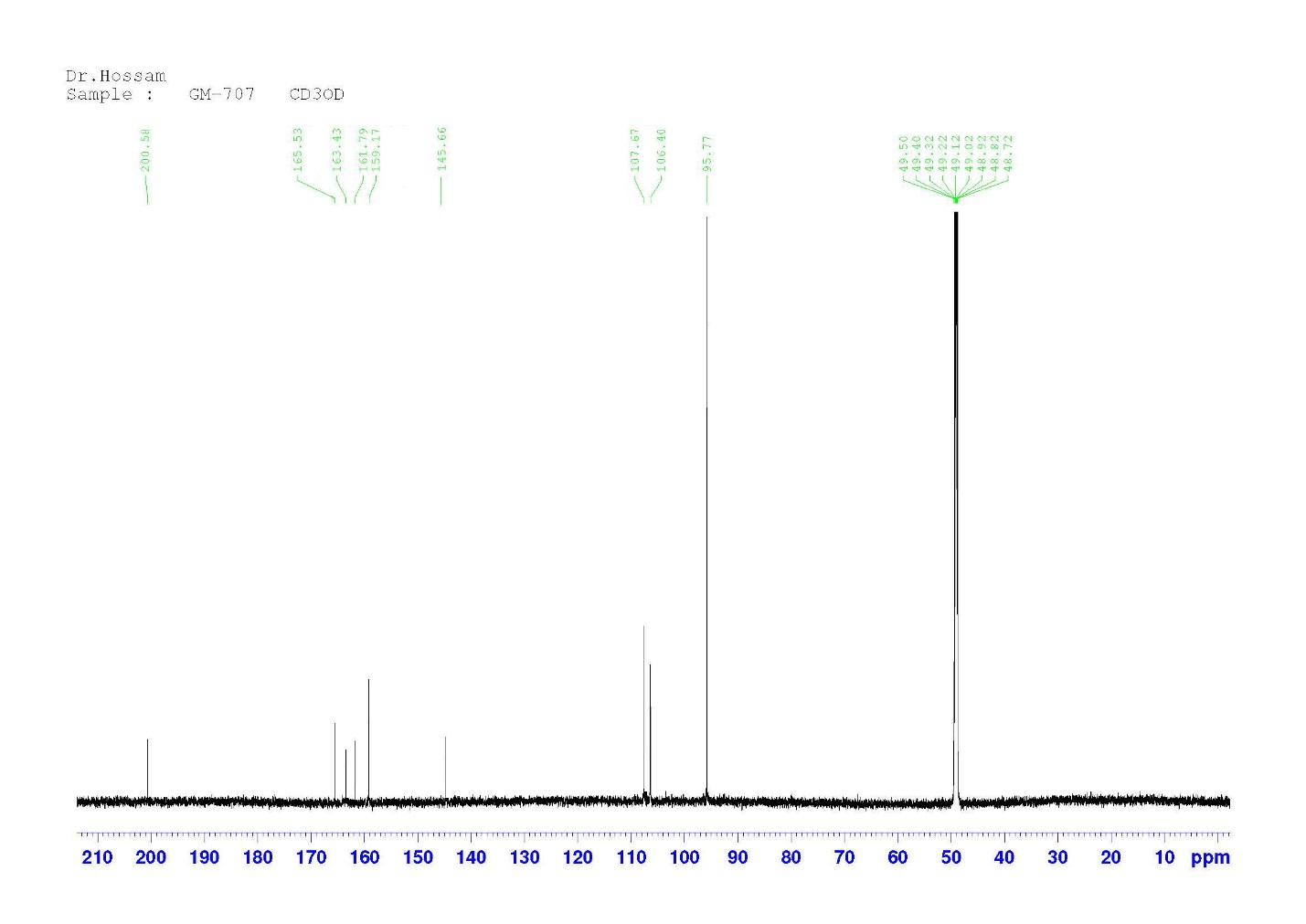
**

**Fig. S12.** ^13^C NMR spectrum of compound **6** (CD_3_OD, 100 MHz).
